# A novel experimental approach for the selective isolation and characterization of human RNase MRP

**DOI:** 10.1080/15476286.2022.2027659

**Published:** 2022-02-06

**Authors:** Merel Derksen, Vicky Mertens, Eline A. Visser, Janine Arts, Wilma Vree Egberts, Ger J. M. Pruijn

**Affiliations:** Department of Biomolecular Chemistry, Institute for Molecules and Materials (IMM), Radboud University, Nijmegen, The Netherlands

**Keywords:** RNase MRP, RNase P, ribonucleoprotein, RNA aptamer

## Abstract

RNase MRP is a ribonucleoprotein complex involved in the endoribonucleolytic cleavage of different RNAs. Mutations in the RNA component of the RNP are the cause of cartilage hair hypoplasia. Patients with cartilage hair hypoplasia are characterized by skeletal dysplasia. Biochemical purification of RNase MRP is desired to be able to study its biochemical function, composition and activity in both healthy and disease situations. Due to the high similarity with RNase P, a method to specifically isolate the RNase MRP complex is currently lacking. By fusing a streptavidin-binding RNA aptamer, the S1m-aptamer, to the RNase MRP RNA we have been able to compare the relative expression levels of wildtype and mutant MRP RNAs. Moreover, we were able to isolate active RNase MRP complexes. We observed that mutant MRP RNAs are expressed at lower levels and have lower catalytic activity compared to the wildtype RNA. The observation that a single nucleotide substitution at position 40 in the P3 domain but not in other domains of RNase MRP RNA severely reduced the binding of the Rpp25 protein subunit confirmed that the P3 region harbours the main binding site for this protein. Altogether, this study shows that the RNA aptamer tagging approach can be used to identify RNase MRP substrates, but also to study the effect of mutations on MRP RNA expression levels and RNase MRP composition and endoribonuclease activity.

## Introduction

**Figure 1.**
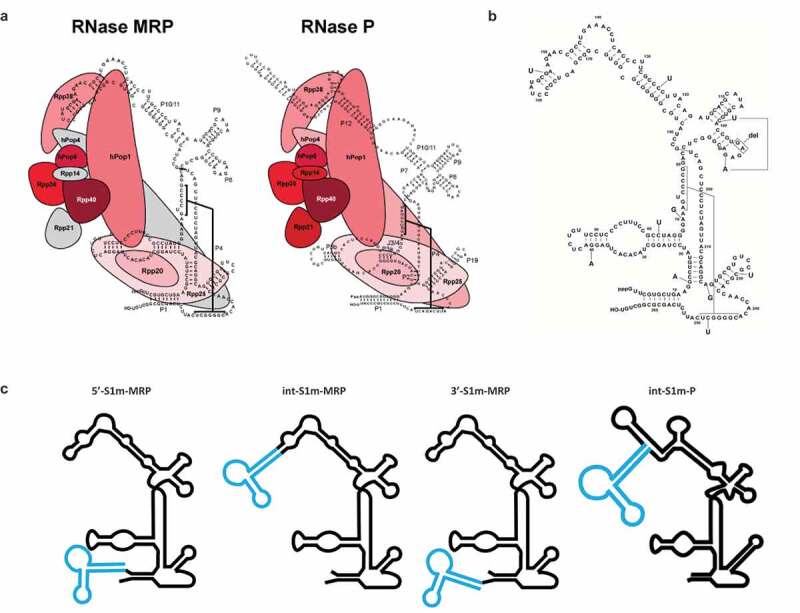
Schematic structures of human RNase MRP and RNase P. (a) Secondary structure of the RNase MRP and P RNAs with the relative positions of the protein subunits based on intermolecular interaction data. It is unclear whether Rpp14, Rpp21 and hPop4 (depicted in grey) stably bind to the RNase MRP complex. Figure adapted from Welting et al [1]. (b). RNase MRP mutations used in this study. (c) The S1m aptamer (blue) is fused to either the 5’- and 3’-end of the MRP RNA (black) or inserted internally (int) between nucleotides 158 and 159. In case of the RNase P RNA the S1m-aptamer was inserted internally between nucleotides 209 and 210.

RNase MRP is a ribonucleoprotein (RNP) consisting of a highly structured RNA molecule (MRP RNA) and multiple protein subunits. The nucleolar RNP is structurally very similar to the precursor tRNA (pre-tRNA) processing complex RNase P (Figure 1(a)). RNase MRP shares its known protein subunits with RNase P, and even though the RNA components differ in sequence and length they fold partly in similar structures [1,2]. Both complexes are endoribonucleases, involved in the cleavage of multiple RNAs. RNase MRP is known to cleave cyclin B2 mRNA, the precursor of 5.8S rRNA and the viperin mRNA [3–6].

The MRP RNA is encoded by the *RMRP* gene, which is transcribed by RNA polymerase III resulting in a 267 nucleotide transcript. The RNP contains at least seven protein subunits (hPop1, Rpp40, Rpp38, Rpp30, Rpp25, Rpp20 and hPop5, Figure 1(a)). Two other proteins, Rpp21 and hPop4 bind *in vitro* to the MRP RNA, but evidence for their stable association in human cells is lacking [7]. For the RNase P subunit Rpp14 neither *in vitro* binding nor *in vivo* association with MRP RNA has been reported [1,7].

Mutations in the MRP RNA are the cause of the autosomal recessive disease Cartilage Hair Hypoplasia (CHH, OMIM #250250). CHH is rarely observed in the general population but occurs with a relatively high incidence in the Amish (1.5:1,000) and Finnish (1:18,000–23,000) populations. Patients affected with CHH are characterized by skeletal dysplasia, leading to short-limbed dwarfism, and hypoplastic hair. CHH is a pleiotropic disease and other symptoms, such as immunodeficiency, Hirschsprung’s disease, childhood anaemia and predisposition to specific cancers, are also frequently observed.

Multiple mutations causing CHH have been reported, and these can be divided in two groups [8]. The first group consists of mutations in the promoter region of *RMRP*. These mutations localize between the TATA box and the transcription start site of RMRP and include duplications, triplications and insertions. Mutations in the promoter region of RMRP lead to reduced transcription and decreased levels of MRP RNA [9]. Promoter mutations in both alleles are never observed, possibly because of embryonic lethality. The second group of mutations is found in the MRP RNA body and include substitutions, small deletions and insertions. This group includes the most frequently observed mutation, the 70A>G substitution, which is close to the presumed catalytic centre of the MRP RNA and might directly affect cleavage activity [10].

The effect of CHH-mutations on the MRP RNA expression in CHH-patients has been described for a small number of patients [3,9,11]. In these patients, reduced MRP RNA expression levels were observed. However, for many other mutations information on the *in vivo* MRP RNA levels is missing. For some mutations, it was demonstrated that they affect the cleavage of the cyclin B2 mRNA or the precursor 5.8S rRNA substrates in human cell culture, whereas others lead to a decreased binding of a subset of the RNP protein components [3,12,13].

Even though a number of CHH-mutations have been studied on RNA expression levels, substrate cleavage and protein binding, a comprehensive overview of the effect of CHH-mutations on MRP RNA expression and stability, RNP composition and cleavage activity is lacking. Currently, no tool is available to isolate active RNase MRP complexes. Due to the high similarity to RNase P, immunoprecipitation of RNase MRP leads to a mixture of these two RNases. Therefore, we developed a method to specifically isolate RNase MRP, using the RNA streptavidin aptamer tag, S1m [14]. Furthermore, we studied the effect of several CHH-mutations in the RNase MRP RNA on the expression level and endoribonucleolytic activity, and on the binding of the Rpp25 protein subunit.

## Materials and methods

### Construction of plasmids

All PCR reactions were performed using the Phusion HSII polymerase (Thermo Scientific) in a two-step protocol. Oligonucleotides used are listed in Supplemental Table 1.

The S1m aptamer sequence, flanked by 5’-BamHI and 3’-BglII restriction sites, was made as described previously [14]. PCR mutagenesis was used to remove the BglII restriction site in the pcDNA5/FRT/TO vector (Thermo Fisher). To prevent overexpression of MRP (and P) RNA, the CMV promoter in the pcDNA5 vector was replaced by restriction digestion and ligation using the MluI and XhoI restriction sites by a doxycycline inducible RNA polymerase III promoter (H1-O2-US) [15], containing a BglII site at the 3’-end. By PCR-based mutagenesis flanking BglII and XhoI sites were introduced in the RMRP DNA sequence at positions corresponding to the 5’ and 3’ termini of RMRP, respectively. Subsequently, a 5’-, 3’- or internal BamHI restriction site was added to the RMRP DNA sequence (Supplemental Figure 1). The resulting PCR products were digested with BglII and XhoI and ligated into the pcDNA5_H1-O2-US vector. Subsequently, the S1m aptamer was ligated in each of the BamHI restriction sites (5’-, internal or 3’- of RMRP and RNase P DNA sequences), resulting in pcDNA5_H1_S1m-MRP and pcDNA5_H1_S1m-P constructs.

MRP RNA mutations were introduced into the S1m-tagged DNA constructs by PCR-based mutagenesis.

### Cell culture

Adherent HEK293T cells were cultured in DMEM supplemented with penicillin/streptomycin and 10% FBS. Transfections were performed 18 to 24 hours after seeding of the cells once the cells reached 20% confluency. For transfection, branched polyethylenimine (PEI):DNA complexes were used in a 1:3 w/w ratio. For the transfection of a single well of a 12 well-plate, 1 µg of DNA was used and 48 hours after transfection the cells were harvested by trypsinization. For the transfection of cells cultured in petri dishes the amounts were adapted in accordance with the surface areas.

### Purification and composition of S1m-tagged RNPs

In order to purify the S1m-tagged RNPs for determining the RNP composition and activity, HEK293T cells in three 15 cm petri dishes were transfected and lysed after 48 hours by sonication in 2 mL of lysis buffer (50 mM Tris-HCl, pH7.6, 10 mM MgCl_2_, 100 mM KCl, 10% glycerol, 0.1% NP-40 and Complete protease inhibitor (Roche)). To remove insoluble material the lysate was centrifuged for 11,000 x g at 4°C for 10 minutes. Subsequently, the lysate was diluted twice in lysis buffer without NP-40, resulting in a final NP-40 concentration of 0.05%. The lysate was added to 40 μL of packed pre-equilibrated streptavidin-Sepharose beads (GE healthcare) and allowed to bind for 16 hours at 4°C. Subsequently, non-bound material was collected and the beads were washed three times with lysis buffer containing 0.05% NP40.

For Western blotting analysis of the S1m-RNase MRPs 0.6% of the input and non-bound material and 10% of the bound material were separated by SDS-PAGE. In case of the S1m-RNase P 3% of the bound material was loaded on the gel. For northern blot hybridization 1% of the input and non-bound material and 6% of the bound material were separated for both S1m-RNase MRP and S1m-RNase P by electrophoresis in a 6% polyacrylamide/8 M urea gel.

### Activity assays RNase MRP and RNase P

For the viperin and pre-tRNA cleavage assays 28% and 9%, respectively, of the wildtype S1m-RNPs bound to the streptavidin beads were used. When comparing the activity of wildtype S1m-MRP to that of mutant S1m-MRP, bound S1m-RNAs to the streptavidin beads were first analysed by northern blotting and the intensities of (mutant) MRP RNA signals were used to quantify the relative amounts of bound material. For the activity analyses of mutant S1m-RNAs equivalent amounts of mutant S1m-RNA were used.

As a positive control a mouse monoclonal anti-Rpp20 antibody (1F11, Modiquest Research) was coupled to protein-A-agarose beads, followed by immunoprecipitation of RNase MRP/P from a HEK293T cell lysate, as described previously [1].

The ^32^P-labeled viperin Vip 401–500 mRNA fragment and precursor tRNA were generated by *in vitro* transcription, as previously described [6]. Briefly, RNA substrates were *in vitro* transcribed using T7 RNA polymerase in the presence of ^32^P-labeled UTP. The viperin substrate was transcribed from a PCR product generated from a plasmid containing the human viperin cDNA. For the tRNA substrate, a linearized plasmid with yeast tRNA^Ser^ was used as a template for in vitro transcription. Beads containing S1m-RNPs or anti-Rpp20 immunoprecipitated material were incubated with the radiolabeled substrates in a buffer containing 20 mM Tris-HCl, pH 7.6, 10 mM MgCl_2_, 50 mM KCl, 1 mM DTT, 50 μg/mL BSA, 16.7 μg/mL *E. coli* tRNA and 4.2 Units/mL RNasin for 1.5–2 hours at 37°C for both the pre-tRNA and the viperin mRNA fragment substrate. The cleavage reactions were stopped by the addition of Trizol, the RNA was isolated, and the reaction products were analysed by electrophoresis in an 8% polyacrylamide/8 M urea gel followed by visualization by phosphorimaging.

### Northern blot hybridization

Cells were seeded in 12 well plates and transfected with (mutant) S1m-tagged RNase MRP constructs. After 48 hours the cells were harvested and total RNA was isolated using Trizol reagent (Ambion). Ten μg of total RNA was fractionated by electrophoresis in a 6% polyacrylamide/8 M urea gel and blotted on Hybond N^+^. After separation on a denaturing polyacrylamide gel, RNA was transferred to Hybond N+ membranes by electroblotting in 18.4 mM NaH_2_PO_4_ and 6.5 mM Na_2_HPO_4_ buffer. After drying the blot, RNA was fixed to the membrane by UV-irradiation at 700 mJ/cm^2^. Blots were blocked at 65°C in pre-hybridization buffer (6x SSC, 0.1 mg/mL sheared herring sperm DNA, 0.2% SDS, 10x Denhardt’s) for one hour. After blocking, radiolabeled antisense probes were added, followed by an overnight incubation. Subsequently the blots were washed twice with 1x SSC, 0.2% SDS and, depending on the remaining radioactivity, once or twice with 0.1x SSC. The signals were detected using Phosphor screens and visualized with the Typhoon imager. Expression levels of the S1m-tagged MRP RNAs were normalized to that of the U3 small nucleolar RNA (snoRNA). Within each experiment the level of the wild-type S1m-MRP RNA was set at 100%. Expression levels of 5’-S1m-MRP RNAs (except 154 G > U) were measured in quadruplicate and that of the 3’-S1m-MRP RNAs in triplicate.

Radiolabeled antisense probes for RNase MRP RNA, RNase P RNA and the U3 RNA were generated by *in vitro* transcription of linearized plasmids as described elsewhere [7].

### Western blotting

After separation by SDS-PAGE, proteins were transferred to nitrocellulose membranes by electroblotting. The blots were incubated with antibodies as described previously [16–20]. The following primary antibodies were used: rabbit polyclonal anti-hPop1 (1:8,000), rabbit polyclonal anti-Rpp40 (1:200), rabbit polyclonal anti-Rpp25 (1:200), rabbit polyclonal anti-Rpp38 (1:200), rabbit polyclonal anti-Rpp30 (1:200), rabbit polyclonal anti-hPop4 (1:100) and mouse monoclonal anti-U1A/U1B” (1:10). The polyclonal rabbit sera against hPop1, Rpp40, Rpp25, Rpp38 and Rpp30 were a kind gift from Sidney Altman and Nayef Jarrous. The specificity of these antibodies was demonstrated by Western blotting (see Supplemental Figure 2). IRDye-conjugated secondary antibodies (LiCOR Biosciences) were used for visualization of bound antibodies. Blots were scanned using the Odyssey Imaging System (LiCOR Biosciences).

## Results

### Purification of S1m-tagged RNase MRP and RNase P complexes

Since all known protein components of human RNase MRP are shared with RNase P, the available antibodies do not allow the selective isolation of either RNase MRP or RNase P. In order to study the effect of CHH-mutations on the protein composition of the RNase MRP complex, we developed an isolation procedure specific for RNase MRP based on the RNase MRP RNA component.

Previously, Li and Altman introduced a streptavidin-binding RNA aptamer (S1) into the RNase P RNA to isolate active RNase P complexes from HeLa cells [21]. Likewise, we introduced the S1 aptamer in the MRP RNA at a similar position to that used for the RNase P RNA, based on the (predicted) secondary structures of these RNAs. In our hands, MRP RNA carrying the S1 tag between nucleotides 158 and 159, when expressed in HEK293T cells, did not bind to streptavidin beads under various conditions (data not shown). We hypothesized that the S1 aptamer was improperly folded or inaccessible due to binding of proteins to or close to the S1 aptamer. To overcome these problems, two changes were made; first, the streptavidin-binding aptamer was also introduced at other positions of the RNase MRP RNA, the 5’- and 3’-ends; second, the S1 tag was replaced by the S1m tag, because S1m has a slightly higher binding affinity for streptavidin (Kd = 29 nM, whereas a Kd of 79 nM was reported for the S1 tag) [14]. This resulted in three versions of S1m-MRP RNA: 5’-S1m-MRP, int-S1m-MRP and 3’-S1m-MRP (Figure 1(b)). To allow a comparison with RNase P, the S1m aptamer was also introduced in the RNase P RNA sequence (at an internal position, Figure 1(b)).

HEK293T cells were transiently transfected with the S1m-tagged RNase MRP and P RNAs constructs and the expressed RNAs were isolated using streptavidin beads. All three S1m-tagged RNase MRP RNAs as well as S1m-tagged RNase P RNA were successfully isolated from the cell lysates (Supplemental Figure 3). Moreover, Western blot analysis of the isolated material showed that at least a subset of the RNase MRP/P proteins were associated with the MRP and P RNAs (Supplemental Figure 4). In case of RNase P, all six of the ten known protein subunits that were analysed (hPop1, Rpp40, Rpp38, Rpp30, hPop4 and Rpp25) were found to bind to the S1m-P RNA. In case of RNase MRP, hPop1 and Rpp25 associated with the S1m-MRP RNAs and also weak signals were observed for Rpp38 in the material isolated with 5’- and 3’-S1m-tagged MRP RNAs. Interestingly, Rpp38 only appeared to associate with the MRP RNAs carrying the S1m tag at the 5’ or 3’ terminus, but not with the internally tagged RNA. As a negative control, an antibody reactive with the U1 and U2 snRNP-associated proteins U1A and U2B” was used. These proteins were indeed not detectable in the material isolated with the S1m-tagged RNAs.

### Endoribonuclease activity of the S1m-tagged RNase MRP and RNase P complexes

**Figure 2.**
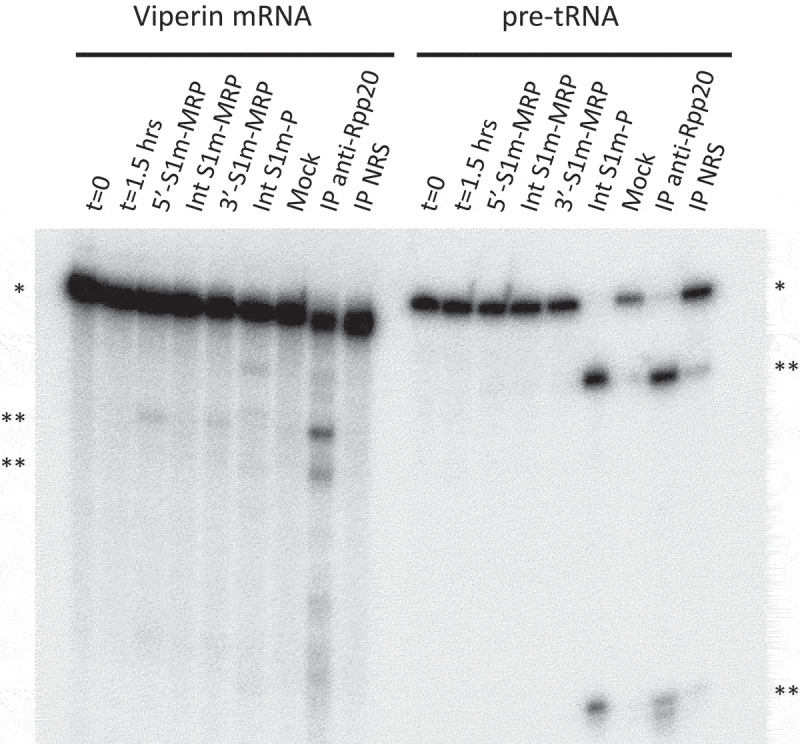
Ribonuclease activity of S1m-MRP and S1m-P complexes. A radiolabeled viperin mRNA fragment and pre-tRNA were incubated for 1.5 hrs with purified S1m-MRP and S1m-P complexes as indicated. The input material and the substrate incubated for 1.5 hrs in the absence of purified material are indicated by t = 0 and t = 1.5 hrs, respectively. Mock refers to the incubation of the substrates with material isolated from a cell lysate of untransfected cells using the same procedure as for the lysates from S1m-RNA expressing cells. Immunoprecipitated material (IP, with an antibody to Rpp20) was used as a control for affinity-purified RNase MRP and RNase P. NRS: normal rabbit serum. * and ** mark the positions of the substrate RNAs and their major cleavage products, respectively.

To investigate whether the complexes formed with the S1m-tagged RNase MRP and RNase P RNAs were functionally active, we incubated the isolated complexes with established substrates for both enzymes, a viperin mRNA fragment for RNase MRP and pre-tRNA for RNase P. The viperin mRNA fragment appeared to be cleaved by the 5’- and 3’-S1m-tagged RNase MRP complexes, but not by the internally tagged complex. Likewise, the pre-tRNA substrate was, as expected, specifically cleaved by the S1m-RNase P complex (Figure 2). Incubation of the viperin mRNA fragment with the S1m-tagged RNase P complex also resulted in a few cleavage products, which, however, are distinct from the products observed with RNase MRP. The immunoprecipitate obtained with an anti-Rpp20 antibody, which was used in parallel as a positive control, resulted in all cleavage products observed with the individual RNase MRP and RNase P complexes. The relatively low efficiency of cleavage of the viperin mRNA fragment by RNase MRP is in agreement with previous observations [6]. Moreover, in the transiently transfected cells the S1m-tagged RNA will compete with the endogenous RNase MRP RNA in ribonucleoprotein particle assembly, which may at least in part explain why the cleavage efficiency with the anti-Rpp20 immunoprecipitate is higher. However, although the signals are weak, they were reproducibly observed in multiple experiments. These results show that the complexes that are assembled on the S1m-tagged RNase MRP RNA (5’ and 3’ tags) and RNase P RNA are functionally active and show substrate specificity.

### Expression levels of mutant S1m-MRPs

The assembly of functional RNase MRP complexes on S1m-tagged MRP RNA in transfected HEK293T cells indicates that this system can be used to assess the effects of mutations on biochemical properties of RNase MRP in an RNase P-independent fashion. It is also important to note that due to the insertion of the S1m-tag the size of the RNA is increased, which means that northern blotting of RNAs extracted from transfected cells allows discriminating between the endogenous MRP RNA and the S1m-tagged exogenous RNA.

**Figure 3.**
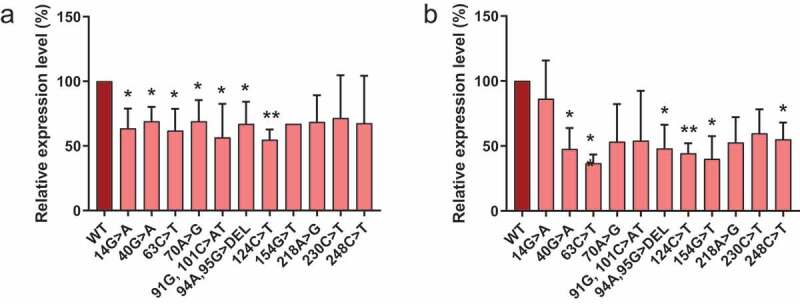
Expression levels of S1m-tagged MRP RNA mutants. HEK293T cells were transfected with S1m-tagged wild type (WT) and mutant MRP RNA constructs (indicated by the nucleotide changes) and the expression levels after 48 hours were determined by northern blot hybridization. Expression levels of (a) 5’-S1m-MRP RNAs and (b) 3’-S1m-MRP RNAs. Levels were normalized based on U3 snoRNA levels and the level of wildtype S1m-MRP was set to 100%. A t-test was performed to determine significance * = P < 0.05, ** = P < 0.01.

Eleven different RNase MRP RNA mutations that are found in CHH patients were introduced in the 5’- and 3’-S1m-tagged MRP RNA. Since the internally S1m-tagged MRP RNA forms an inactive complex, we did not pursue analyses with this variant. The selected mutations were spread across the MRP RNA molecule and except for the 70A>G, 94A, 95 G> DEL and 218A>G mutations, all are located in double-stranded regions of the predicted secondary structure. We investigated the effects of these CHH-mutations on the expression level of the RNA. To this end, we expressed the S1m-tagged wildtype and mutant MRP RNAs in HEK293T cells and assessed their expression levels by northern blot hybridization (Figure 3). The results show that for both 5’- and 3’-S1m-tagged MRP RNAs the levels of the mutants are approximately two-fold lower than that of the wildtype MRP RNA. Since the transcription regulatory elements for both the 5’- and the 3’-S1m-tagged RNAs are the same, the differences in levels most likely reflect differences in turnover rates.

### S1m-RNase MRP as a tool to study complex formation and activity

**Figure 4.**
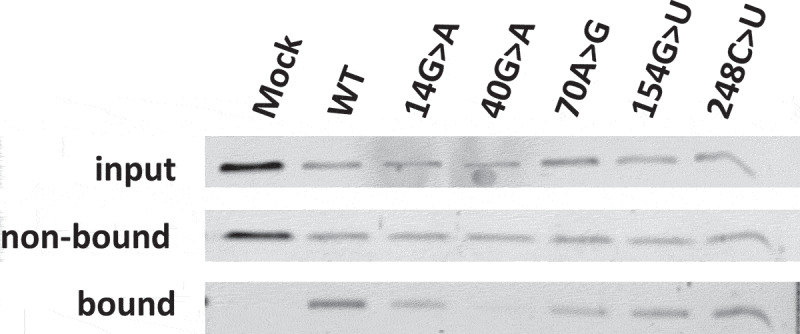
Association of Rpp25 with 3’-S1m-MRP RNAs. 3’-S1m-MRP RNAs (mutants indicated by nucleotide substitutions) were isolated from transfected HEK293T cells using streptavidin beads. Input, non-bound and bound material were analysed by Western blotting using a polyclonal anti-Rpp25 antibody. Equivalent amounts of input and non-bound fractions were loaded. The relative amount of bound material analysed was based on the amount of bound S1m-MRP quantified by northern blotting. Mock refers to material isolated from a cell lysate of untransfected cells.

**Figure 5.**
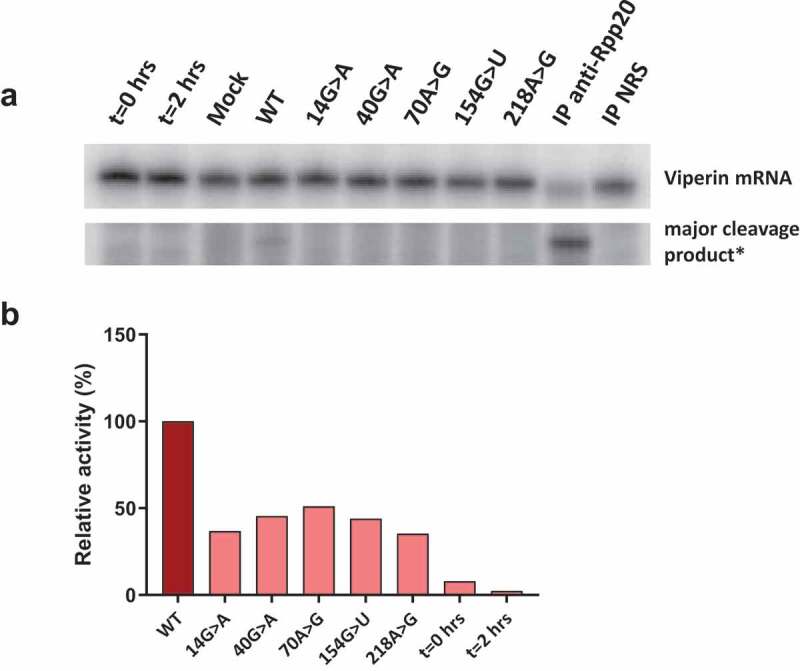
Catalytic activity of wild type and mutant RNase MRP complexes. 3’-S1m-MRP RNA-containing complexes were isolated from transfected cell lysates and incubated with the radiolabeled viperin mRNA fragment. Reaction products were analysed by denaturing gel electrophoresis and autoradiography. A) Substrate RNA and major cleavage products. *Note that the results are from the same gel, but with different exposure times (longer exposure for detection of cleavage product). Material immunoprecipitated (IP) with anti-Rpp20 antibodies and with antibodies from normal rabbit serum (NRS) was used as control. B) Relative cleavage activity observed for the 3’-S1m-MRP complexes. Activity of the wild type (WT) 3’-S1m-MRP complex was set to 100%. Lanes marked t = 0 hrs and t = 2 hrs represent the viperin mRNA substrate incubated for 0 and 2 hours in the absence of isolated material.

To show that isolated S1m-MRPs can be used to study the association of specific proteins with MRP RNA mutants and the enzymatic activity of such mutant RNase MRPs, a selection of 3’-S1m-MRP mutants was used to investigate the binding of Rpp25 and the cleavage of the viperin mRNA fragment substrate. Rpp25 is known to bind to the P3 region (nucleotides 22–67, Figure 1) of the MRP RNA. *In vitro* studies have shown that the 40 G > A substitution leads to reduced binding of Rpp25 to this part of the MRP RNA [13]. The analyses with mutant S1m-tagged MRP RNPs assembled in HEK293T cells demonstrate that the 40 G > A mutation also in this experimental setting severely reduced binding of Rpp25 to the MRP RNA (Figure 4), whereas mutations in other regions of the RNA did not or only marginally affect binding of the Rpp25 subunit. Finally, the activity of these S1m-MRP mutants on the viperin substrate was assessed (Figure 5). We observed that all CHH-mutants analysed led to a decreased cleavage activity for this substrate.

## Discussion

In this study, an RNA-based tagging approach to isolate reconstituted, enzymatically active RNase MRP complexes from human cells has been developed. The fusion of the S1m aptamer to the MRP RNA allows for specific purification of RNase MRP without copurification of the structurally related RNase P complex. The tagged RNase MRP RNAs were shown to form RNPs with at least a subset of RNase MRP proteins (hPop1, Rpp38 and Rpp25). Although not all known protein subunits were confirmed to be associated with the MRP RNAs, the 5’- and 3’-S1m-tagged versions were catalytically active, as demonstrated by viperin mRNA fragment cleavage *in vitro*. The substrate-specificity of these S1m-MRP complexes was established by the lack of pre-tRNA cleavage.

Previously, it was shown that viperin mRNA is cleaved by immunoaffinity purified material obtained with anti-Rpp25 and anti-hPop4 antibodies, which will contain a mixture of RNase MRP and RNase P complexes [6]. More recently, knockdown of RMRP in CHH fibroblasts led to increased levels of viperin suggesting that the viperin mRNA indeed is an RNase MRP substrate [22]. Here, we provide evidence that viperin mRNA is directly cleaved by RNase MRP and not by RNase P. The selective isolation of S1m-tagged MRPs is therefore applicable for the validation of putative RNase MRP substrates *in vitro*. Compared to the cleavage of the pre-tRNA substrate by RNase P, the efficiency by which the viperin mRNA fragment is cleaved by RNase MRP is low. As a consequence of this low efficiency, studying the effect of RMRP mutations on the cleavage activity using this substrate is difficult, especially when quantitative data are desired. For five mutants with CHH-derived mutations at various locations in RMRP virtually no cleavage was observed, but it should be noted that the signals obtained with the wild type RMRP are rather weak, which means that a partial reduction might already lead to signals below the detection threshold. Nevertheless, these data are in line with the reduction of catalytic activity of RNase MRP by CHH-associated mutations in the RNA component.

The low cleavage activity of the 5’- and 3’-S1m-MRPs may be explained by the inefficient association of some protein subunits. It is possible that the S1m aptamer interferes with the binding of some proteins. Although the function of the individual protein subunits is unknown, they might be important for the catalytic activity of the complex. From a more quantitative point of view, it should be noted that transiently transfected cells expressing the S1m-tagged RNAs do not provide meaningful information on the ratio between the tagged and corresponding endogenous RNAs. Based upon northern blot hybridization signal intensities obtained with data from several experiments, the levels of tagged RNAs were estimated to be similar to those of the endogenous RNAs. However, it should be taken into account that during transient transfection only a subset of cells will express the transgene and that the levels of transgene expression may differ between individual cells. Therefore, the generation of stably transfected cell lines is required for more quantitative analyses.

Also, the immunoprecipitated RNase MRP/P complexes displayed a relatively low cleavage activity for the viperin mRNA fragment when compared to cleavage of pre-tRNA. In addition to suboptimal particle assembly, this may also be explained by differences in the catalytic activity between RNase MRP and RNase P or by suboptimal folding of the viperin mRNA fragment. Recently, RNase MRP has been implicated in the cleavage of mRNAs containing methylated adenosine, m6A, the most abundant internal modification in RNA [23]. Endoribonucleolytic cleavage of m6A-containing RNAs appeared to be mediated by an m6A-reader protein, an adaptor protein and RNase MRP/RNase P. Therefore, it is tempting to speculate that the efficiency by which the viperin mRNA is cleaved *in vivo* is dependent on methylation of the RNA. To more accurately quantify the effects of RMRP mutations on the cleavage activity, a substrate that is more efficiently converted by S1m-tagged RNase MRP is highly desired.

The internally S1m-tagged RNase MRP did not show any *in vitro* cleavage activity. Nevertheless, the MRP RNA can be isolated using streptavidin beads and appeared to be bound by hPop1 and Rpp25, just like the 5’- and 3’-S1m-tagged MRP RNAs. Although in theory the S1m-tag at the internal position might interfere with the enzymatic activity, it is more likely that improper particle assembly is responsible for this phenomenon. Indeed, Rpp38 did not detectably associate with the reconstituted complex, in contrast to the particles that were formed when the S1m-tag was positioned at the 5’- or 3’-end of RMRP. Since the internal tag was inserted in a region that is known to be involved in Rpp38 association [7], the tag probably interferes with the binding of this protein.

The generation of S1m-tagged MRPs was aimed at studies on the effects of mutations on the biochemical properties of this complex. We have shown that this approach not only allows the assessment of expression levels, protein binding and cleavage activity but that also the effects of mutations can be studied. The majority of the CHH-related RMRP mutants were expressed at significantly lower levels compared to the wildtype RNA. It is not yet known whether the lower expression levels are due to decreased RNA stability or transcription efficiency, but it is likely that the mutations destabilize the RNA. The binding of Rpp25 to the mutants was as expected based on the previously determined interaction between Rpp25 and the P3 region and therefore the 3’-S1m-tagged MRP provides a tool to analyse the binding of proteins to RMRP mutants, at least for Rpp25, Rpp38 and hPop1.

We conclude that the use of the S1m-aptamer leads to successful isolation of RNase MRP. The method we developed represents the first experimental approach that allows for the highly selective purification of human RNase MRP in the absence of RNase P. This selectivity of S1m-MRPs isolation greatly facilitates the validation of putative RNase MRP substrates *in vitro*, as shown by the cleavage of viperin mRNA. Furthermore, the S1m-MRP complexes can be used to study the effects of CHH-associated RMRP mutations on its expression level, RNase MRP protein composition and the enzymatic activity.

## Supplementary Material

Supplemental Material

## Data Availability

Data is available from the corresponding author upon request.
