# Supplementary material for: A novel experimental approach for the selective isolation and characterization of human RNase MRP
Source: RNA Biol. 2022 Feb 6;19(1):305–12. doi: 10.1080/15476286.2022.2027659 (PMC8820802; doi:10.1080/15476286.2022.2027659)
Supplement: Supplemental Material [file KRNB_A_2027659_SM7581.zip › supplementary/SUPPL DTATA.pdf]

Supplementary data

A novel experimental approach for the selective isolation and characterization of human RNase MRP

Merel Derksen, Vicky Mertens, Eline A. Visser, Janine Arts, Wilma Vree Egberts, Ger J.M. Pruijn[1]

Supplemental Table 1: Oligonucleotides used in this study.

Used primer pairs	Sequence (5' → 3')[a]	Purpose
pcDNA5-BglII-Rem_Fw	AGATCACCCGATCCCCTATGGTGCACTC	Destruction of BglII site on pcDNA5/FRT/TO vector
pcDNA5-BglI-Rem_Rv	GGGGATCGGGTGATCTCCCGATCCGTCGACGTC	
Int-BamHI-P_Fw	CTAGTAGGATCCATGATCGGGGCCCGGCGGATG	Introduction internal BamHI site into the RNase P RNA (between nucleotides 205-206)
Int-BamHI-P_Rv	GATCATGGATCCTACTAGCCCCGAAGGGCGGGG	
5'-BglII-P_Fw	GACCACAGATCTCCATGGGCGGAGGGAAGC	Introduction 5'-BglII and 3'-XhoI site flanking RNase P RNA (with internal BamHI site)
3'-XhoI-P_Rv	CCCCCTCGAGTAAAAAAATGGGCGGAGGAGAGTAG	
5'-BglII-BamHI-MRP_Fw	CTAGTAAGATCTCCGGATCCGTTCGTGCTGAAGGCCTG	Introduction 5'-BamHI in RNase MRP RNA flanked with 5'-BglII and 3'-XhoI sites.
3'-XhoI-MRP_Rv	TGGTCTCCTCGAGTAAAAAAACAGCCGCGCTGAGAATG	
Int-BamHI-MRP_Fw	CTAGTAGGATCCATGATCTCCCGCTGAGCGGC	Introduction Internal BamHI site into RNase MRP RNA (between nucleotides 158-159)
Int-BamHI-MRP_Rv	GATCATGGATCCTACTAGTACGCTTCTTGGCGGACTTTG	
5'-BglII-MRP_Fw	AGATCTCCGTTCGTGCTGAAGGCCTG	Introduction 5'-BglII and 3'-XhoI site flanking RNase MRP RNA (with internal BamHI site)
3'-XhoI-MRP_Rv	TGGTCTCCTCGAGTAAAAAAACAGCCGCGCTGAGAATG	
5'-BglII-MRP_Fw	AGATCTCCGTTCGTGCTGAAGGCCTG	Introduction 3'-BamHI site into RNase MRP RNA
3'-XhoI-BamHI-MRP_Rv	CTAGTACTCGAGAAAAAAAGGATCCACAGCCGCGCTGAGAATG	
14G>A_Fw	GAAGACCTGTATCCTAGGCTACACACTGAGG	SDM WT-MRP; 14G>A
14G>A_Rv	CCTAGGATACAGGTCTTCAGCACGAAC	
40G>A_Fw	CACACTGAGAACTCTGTTCCTCCC	SDM WT- MRP; 40G>A
40G>A_Rv	GGAACAGAGTTCTCAGTGTGTAGCC	
63C>T_Fw	CTTTCCGCTTAGGGGAAAGTCCC	SDM WT-MRP; 63C>T
63C>T_Rv	CCTAAGCGGAAAGGGGAGGAAC	
70A>G_Fw	GCCTAGGGGGGAAGTCCCCGGAC	SDM WT-MRP; 70A>G
70A>G_Rv	GGGACTTCCCCCTAGGCGGAAAG	
91G>A_Fw	GGGCAAAGAGTGCCACGTGCATAC	SDM WT-MRP; 91G>A
91G>A_Rv	CACTCTTTGCCCGAGGTCCG	
101C>T_Fw	GTGCCATGTGCATACGCACGTAGAC	SDM MRP91G>A; 101C>T
101C>T_Rv	GTATGCACATGGCACTCTTTGCCC	
94,95 DEL AG_Fw	GGCAGAGTGCCACGTGCATACG	SDM WT-MRP; 94,95 DEL AG
94,95 DEL AG_Rv	GTGGCACTCTGCCCGAGGTC	
124C>T_Fw	GTAGACATTCTCCGCTTCCCACTCC	SDM WT-MRP; 124C>T
124C>T_Rv	AGCGGAGAATGTCTACGTGCGTATG	
154G>T_Fw 5'!	CCAAGAATCGTATCCCCGCTGAGC	SDM WT-MRP; 154C>T
154G>T_Rv 5'!	GTACGATTCTTGGCGGACTTTGGAG	
218A>G_Fw	GTTACGCAGGCGGTGCGTG	SDM WT-MRP; 218A>G
218A>G_Rv	CACGCACCGCCTGCGTAAC	
230C>T_Fw	GTCCGTGCACCAACCACACG	SDM WT-MRP; 230C>T
230C>T_Rv	GGTGCACGGACACGCACTG	
248C>T_Fw	CGGGGTTCATTCTCAGCGC	SDM WT-MRP; 248C>T
248C>T_Rv	GAATGAACCCCGTGTGGTTGG	

[a] Restriction sites are underscored. SDM: site directed mutagenesis.

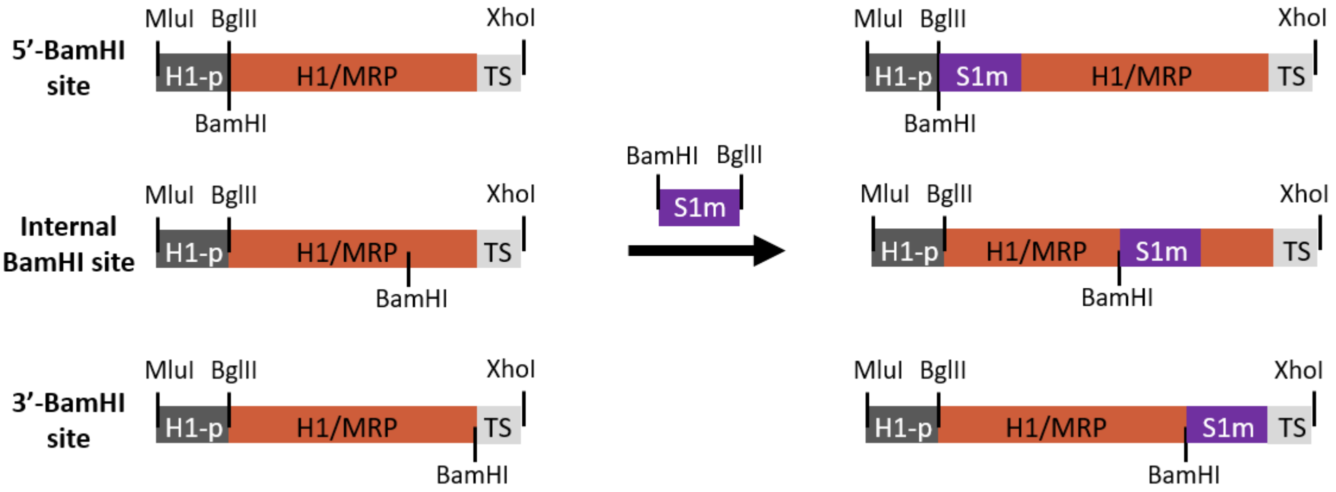

Supplemental Figure 1: Overview of DNA constructs. First pcDNA5 constructs containing the H1_O2_US promoter (H1-p) followed by the RNase P (H1) or MRP RNA sequence with a 5', internal or 3' BamHI site were generated. A oligo-dT termination signal (TS) at the 3'-end was added to ensure transcription termination by RNA polymerase III. Subsequently the S1m (flanked with a 5'-BamHI and 3'-BglII site) was ligated into the BamH1 site of these constructs to obtain the final constructs used for transfections.

Supplemental Figure 2: Western blot analysis of antisera against RNase MRP/RNase P subunits. Whole cell lysates of HEK293T cells were separated by SDS-PAGE and transferred to nitrocellulose membranes. Strips were incubated with polyclonal antibodies against hPop1, Rpp38, Rpp30, hPop4, Rpp40 and Rpp25. Arrowheads mark the positions of the respective RNase MRP/RNase P proteins.

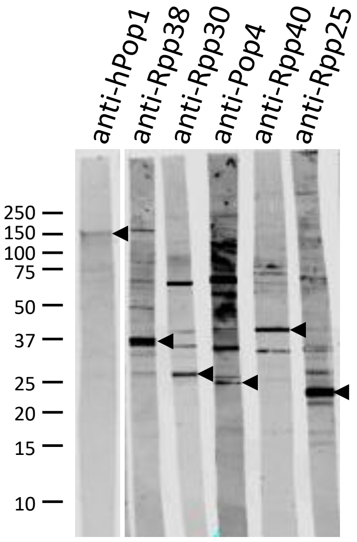

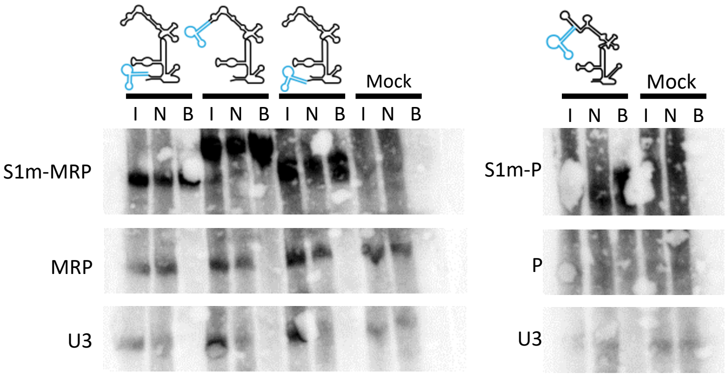

Supplemental Figure 3: Isolation of 5'-, 3'- and/or internally tagged S1m-RNase MRP and S1m-RNase P. HEK293T cells were transfected with S1m-tagged RNA constructs and after 48 hours the S1m-RNAs were isolated from cell lysates using streptavidin beads. RNAs were extracted and analyzed by northern blot hybridization using RNase MRP RNA-, RNase P RNA- and U3 snoRNA-specific probes. For each construct, input (I), non-bound (N) and bound (B) material was analyzed. Mock represents material isolated from a cell lysate of untransfected cells.

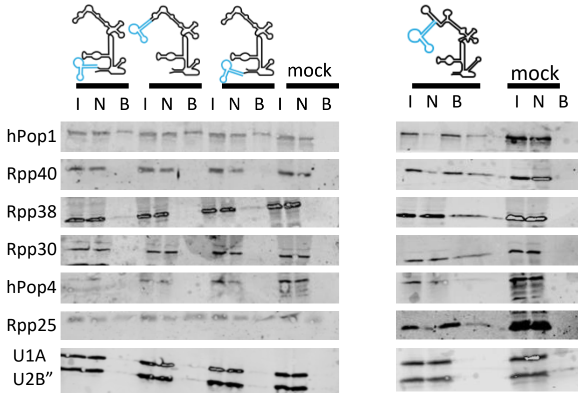

Supplemental Figure 4: Association of protein subunits with S1m-MRP and S1m-P RNAs. S1m-RNAs (S1m-MRP: left panels; S1m-P: right panels) were purified using streptavidin beads and the input (I), non-bound (N) and bound (B) fractions were analysed by western blotting using RNase MRP/P protein-specific antibodies. U1A and U2B" are U1 and U2 small nuclear RNP-associated proteins, which were used as background controls. Mock represents material isolated from a cell lysate of untransfected cells.
